# Prevalence and Patterns of Traumatic Dental Injuries in the Permanent Dentition: A Three-Year Retrospective Overview Study at the University Dental Clinic of Vienna

**DOI:** 10.3390/ijerph192315725

**Published:** 2022-11-25

**Authors:** Sophie Lembacher, Steffen Schneider, Stefan Lettner, Katrin Bekes

**Affiliations:** 1 Department of Paediatric Dentistry, University Clinic of Dentistry, Medical University of Vienna, Sensengasse 2a, 1090 Vienna, Austria; 2 Department of Oral and Maxillofacial Surgery, Medical University of Vienna, Waehringer Gürtel 18-20, 1090 Vienna, Austria; 3 Karl Donath Laboratory for Hard Tissue and Biomaterial Research, Statistics, School of Dentistry, Medical University of Vienna, Sensengasse 2a, 1090 Vienna, Austria

**Keywords:** traumatic dental injuries, permanent teeth, prevalence, Austria, tooth fracture

## Abstract

The aim of this study was to retrospectively analyze the prevalence and patterns of traumatic dental injuries (TDIs) in permanent teeth at the University Dental Clinic of Vienna and examine influential variables. The study included all patients with dental trauma in permanent teeth who presented at the University Dental Clinic of Vienna (Austria) between 2014 and 2016. Dental records, including age, gender, location of trauma, type of trauma, cause of TDI, and location of the traumatic incident, were obtained. Clinical oral and radiographic examinations were conducted in accordance with the current guidelines of the German Society of Dental, Oral, and Craniomandibular Sciences (DGZMK). The sample comprised 1132 permanent teeth of 578 patients with TDIs. The most frequently injured teeth were upper central incisors (n = 719, 63.5%), followed by upper lateral incisors (n = 231, 20.4%). Fractures were the most frequent injury type (53%, n = 596). TDIs mostly occur due to falling accidents. The majority of traumatic incidents occurred at home (79.4%, n = 459). The injury characteristics are comparable to the results of other international studies. Due to the high prevalence of TDIs in dental medicine, dental practitioners should be equipped to effectively manage their immediate care and treat potential long-term complications.

## 1. Introduction

Traumatic dental injuries (TDIs) are one of the main motives for medical consultation in dentistry [1]. Even though the oral region comprises only 1% of the whole body, it accounts for 5% of all physical injuries [2]. Besides facial soft tissue injuries and facial bone fractures, dental trauma is one of the most common injuries in the craniomaxillofacial region [3]. A recent study by Petti et al. analyzed 232 international studies published between 1996 and 2016. The results showed that over one billion people are affected by dental trauma worldwide [4]. The global prevalence is estimated at 6–59% [1]. Approximately 50% of children experience a dental injury before the age of 18 [5]. The highest risk of suffering from TDI in permanent dentition is between the ages of 7 and 12 [2]. As the roots are still developing in this period of time, traumatic injuries may negatively influence further tooth growth. Therefore, the immediate emergency care at the time and place of the traumatic incident and initial professional medical treatment has a serious impact on the prognosis of the traumatized teeth [2,6]. Reports indicate that TDIs and their consequences may exceed the burden of caries and periodontal disease in the young population [7,8]. The severity of injuries ranges from minimal enamel loss to complex fractures with pulp exposure or from subluxations to avulsions [9]. In the future, an increase in dental trauma may be expected as the senior population is more often maintaining their permanent teeth due to the overall improvement of oral health in the past decades [10].

In recent years, extensive research in etiology, diagnostics, treatment, and potential complications of TDI in permanent teeth was conducted. Results show a wide range of frequency rates [1]. This variability arises from differences in study types [1,6], applied classification systems, studied population [10], geographical region, and behavioral or cultural characteristics [11]. 

However, there has been little epidemiological research on dental trauma in Austria. Existing epidemiologic data primarily focuses on dental trauma in the context of facial injuries or is based on selective patient cohorts limited to different injury or accident types in permanent teeth [3,12]. Therefore, the presented article reports the first data on dental trauma in permanent teeth in Vienna. It aims to give the first overview of the prevalence, patterns, and causes of TDIs and to compare the results in an international context. The Department of Emergency Dental Care at the University Dental Clinic of Vienna is the only specialized unit for TDIs in the area within the public university system. In view of 944 local dentist offices, two additional hospitals with dental-care units, and 18 outpatient clinics, the tendency for a decentralized approach regarding the provision of dental care in Vienna, a city with 1.87 million inhabitants, is apparent. Based on existing literature, several variables relating to TDIs were analyzed, including gender, injury type, affected teeth, location of the traumatic incident, and cause of TDIs. The study aims to give a current overview of the regional prevalence, patterns, and etiology of TDIs. The collected data is compared with analyses from other countries regarding gender, teeth affected, injury type, cause of TDI, and location of the traumatic incident. 

## 2. Material and Methods

The study was conducted as a retrospective overview study, including all patients with traumatic dental injuries (TDI) in permanent teeth who presented at the Department of Emergency Dental Care at the University Dental Clinic in Vienna (Austria) between 2014 and 2016. Medical records were retrieved from the patient registry. All patients with TDIs affecting the permanent dentition that sought initial assessment and emergency treatment at the department were included. Exclusion criteria comprised injuries of deciduous teeth or deficient records. Data were classified and grouped according to age, gender, the time interval between trauma and arrival at the clinic, affected teeth, injury type, cause of TDI, and location of the traumatic incident. The classification of dental trauma was conducted in accordance with the classification system of the World Health Organization, which was slightly modified by Andreasen et al. [9]. It is based on distinguishing between injuries to the hard dental tissues and pulp and periodontal tissue. Hence, TDIs were classified into injuries to the dental hard tissues and pulp (enamel infraction, crown fracture without pulp exposure (uncomplicated crown fracture), crown fracture with pulp exposure (complicated crown fracture), crown-root fracture, root fracture) and injuries to the periodontal tissue (concussion, subluxation, lateral dislocation, intrusion, extrusion, and avulsion). In addition to these diagnostic entities by Andreason, this study introduced combined injuries as a third category. Combined injuries were defined as simultaneously occurring fractures and dislocations within the same tooth at the same time (meaning per trauma). Consequently, the main injury types were categorized as fractures, dislocations, and combined injuries (Table 1). Clinical oral and radiographic examinations were conducted in accordance with current guidelines of the German Society of Dental, Oral, and Craniomandibular Sciences (DGZMK).

Statistical analysis was performed with R version 4.0.2 (R Core Team 2020, Vienna, Austria). Descriptive statistics (mean, SD) were provided for continuous measurements (time to treatment, age). Nominal measurements (e.g., gender, type of injury, type of accident) are summarized using frequencies and proportions as well as crosstabulations. Wilson score intervals and χ^2^ tests with continuity correction were calculated to compare interesting proportions. For visualization, kernel density estimators (KDE), a continuous version of histograms, were used. Graphs were created using package ggplot 2 (Wickham, Hadley 2009, New York, NY, USA).

## 3. Results

**Figure 1:**
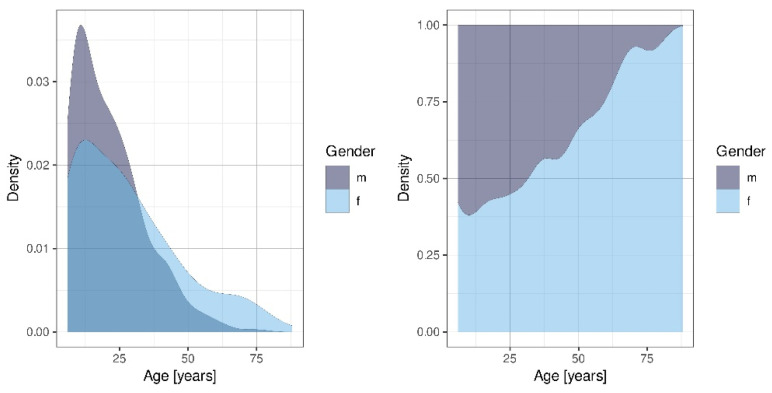
Age distribution of patients with regard to gender.

The sample comprised 1132 permanent teeth of 578 patients with traumatic dental injuries in the permanent dentition. At the time of injury, the mean age was 23.8 years (±16.0, range 6–88 years). Two age peaks were observed. After a peak between and 12 years, the TDI decreased, only to increase again between the ages of 20 and 25 (Figure 1). With 59.17% male (95% CI: 55.0–63.2%, n = 342) and 40.83% female (95% CI: 36.8–45.0%, n = 236) patients being affected, the gender ratio was 1:1.45. With a χ^2^-Test with Yates’ continuity correction calculating a p-value smaller than 0.0001, men were significantly more affected by TDIs than women. Three hundred eighty-two patients (66.1%) immediately presented at the Department of Emergency Dental Care at the University Dental Clinic of Vienna. The other patients were either referrals or no information was available (n = 196, 33.9%). 

**Figure 2:**
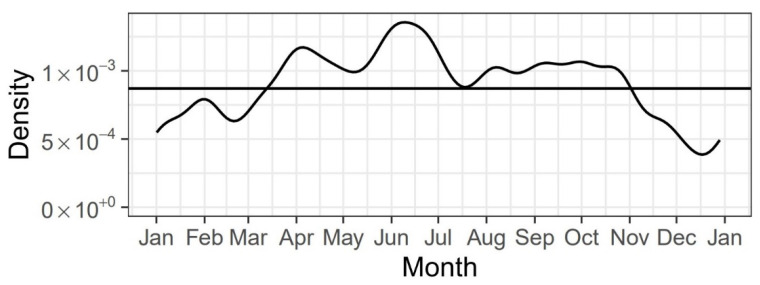
Kernel density estimation (KDE) plot for the relative likelihood of a TDI per date.

**Figure 3:**
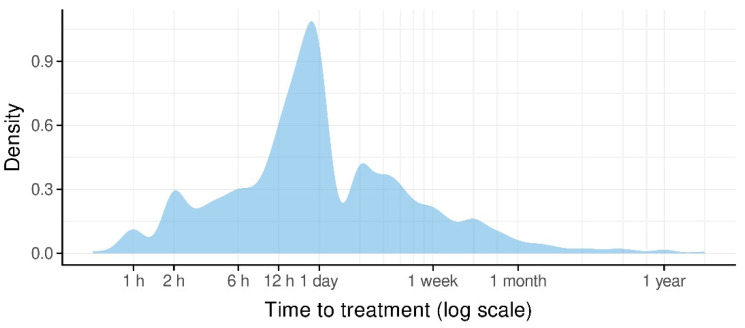
Time to treatment.

The majority of patients presented during official clinic hours on weekdays. However, with 101 and 114 patients on Fridays and Saturdays, these were the busiest days of the week. On the other days, the incidence never surpassed 100 patients (Table 2). Figure 2 shows the relative likelihood of a TDI per date in a kernel density estimation (KDE) plot. The horizontal line shows the average relative likelihood for comparison. From early November to the end of March, there were relatively fewer TDIs. A relatively higher amount of TDIs was observed from April to the beginning of November. With regard to clinic arrival time following dental trauma, 66.0% (n = 356) sought treatment within the first 24 h after TDI with a maximum of 2 h. Figure 3 shows the average time passed until treatment.

**Figure 4:**
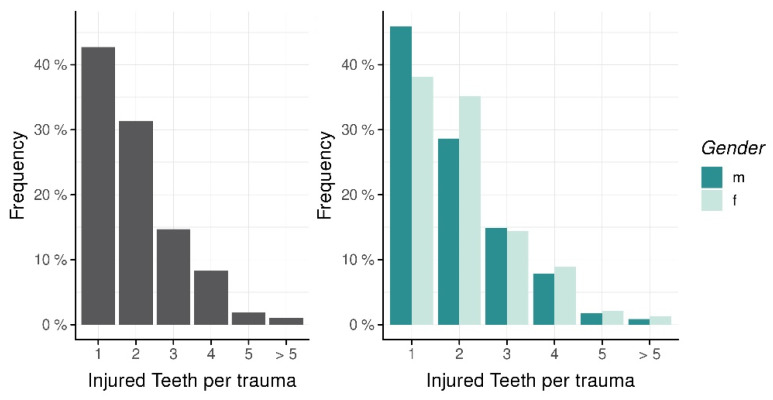
Number of injured permanent teeth per trauma and gender.

**Figure 5:**
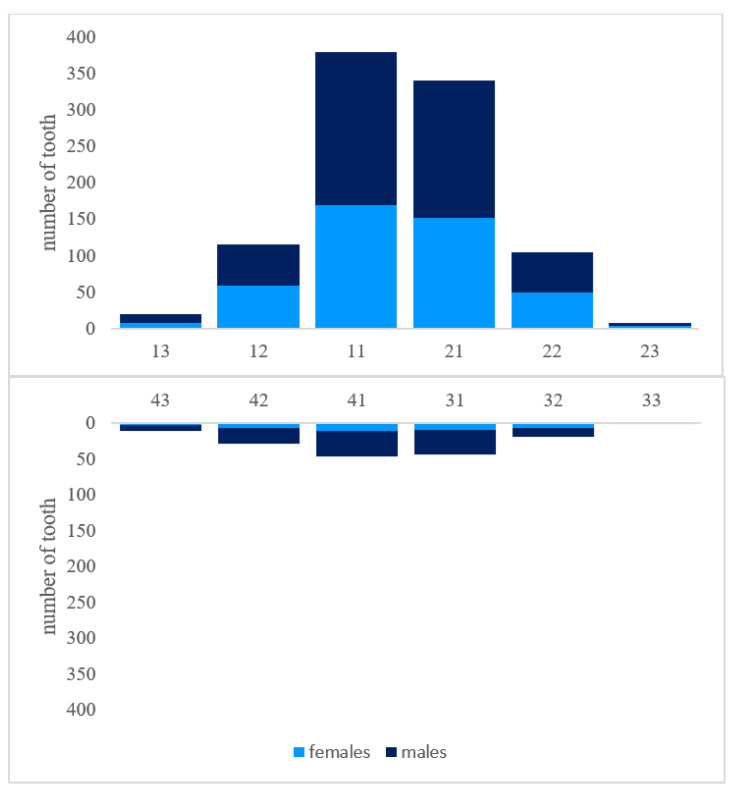
Distribution of traumatized teeth type per gender.

Five hundred sixty-eight (98.3%) patients suffered from dental trauma for the first time, whereas ten patients (1.7%) had previously experienced TDIs. Accompanying soft tissue injuries (laceration, abrasion, etc.) were present in 225 (38.9%) patients. In 42.7% (n = 247) of cases, only one tooth was affected. 31.3% (n = 181) showed two traumatized teeth within the same incident (Figure 4). Upper teeth were significantly more often affected by TDI (n = 496, 85.8%). In 6.7% (n = 39), TDI occurred in the lower jaw, 85.8% in the upper jaw, and 7.4% (n = 43) in the upper and lower jaw at the same time. The most frequently injured teeth were upper central incisors (n = 719, 63.5%), followed by upper lateral incisors (n = 231, 20.4%). Figure 5 summarizes the location of TDI monitored in this report. 85% (n = 960) of TDIs showed isolated types of injuries, whereas 15% (n = 172) were identified as combined injuries. With 53% (n = 596), fractures were the most frequent injury type. The most common types of fractures were uncomplicated crown fractures (no pulp exposure) (64.9%, n = 387), complicated crown fractures (with pulp exposure (15.4%, n = 92), enamel cracks (8.7%, n = 52) and crown-root-fractures (8.1%, n = 48). Three hundred eighty-two patients suffered from dislocations (34%). The three most prevalent subtypes of dislocations were concussions (21.7%, n = 83), subluxations (27.5%, n = 105), and lateral dislocations (26.7%, n = 102). Extrusions (6%, n = 23) were more frequently observed than intrusions (3.9%, n = 15). Fifty-two patients experienced avulsions (13.6%). In 14% (n = 154) of patients, both periodontal hard dental tissue injuries were present. The prevailing majority of combined injuries consisted of a combination of two different injury types. The most frequent combinations were crown fractures with increased tooth mobility (3.2%, n = 36) and crown fractures along with concussion (2.9%, n = 33). In five cases, a combination of three different injury types was observed. Four patients showed crown fractures, concussions, and enamel cracks, and one patient suffered an avulsion combined with crown and root fracture within the same tooth at the same traumatic incident. Table 3 gives an overview of the distribution of fractures, dislocations, and combined injuries. Table 4 provides an incidence matrix, including detailed information on the frequency of TDI occurrence in regard to different injury types. It also shows all possible combinatory variations of injury types. For instance, 34 patients suffered from extrusions. In 30 out of these 34 cases, no other injury was observed. In three cases, however, the extrusion occurred in combination with crown fractures. In one of these cases, even with pulp exposure. Another patient showed an extrusion and root fracture. 

Evaluating different causes of TDIs results show that most dental trauma in permanent teeth was caused by falling accidents (male: n = 189, 55.3%; female: n = 176, 74.6%), followed by punching (male: n = 96, 28.1%; female: n = 41, 17.4%). Fourteen males (4%) and three females (1%) experienced dental trauma due to collision accidents (Table 5). The correlation between accident and injury types established by crosstabulations proved that punching was the most prevalent cause for all injury types (fractures, dislocations, combined injuries). In 79.4% (n = 459) of cases, the traumatic incident occurred at home, in 5.2% (n = 30) in traffic. 9.5% (n = 55) of TDIs were caused by accidents during leisure activities. 0.9% (n = 5) presented as work accidents, 2.4% (n = 14) as accidents at school.

## 4. Discussion

In recent years, traumatic dental injuries have been the focus of extensive research. Preventative measures and accurate treatment were broadly discussed topics among dental professionals. The present study comprised data from patients seeking emergency care at the Department of Emergency Dental Care of the University Dental Clinic of Vienna over three years from 2014 to 2016. 

According to the collected data, males suffered more TDIs than females (m: n = 342, female: n = 236). This correlates with previous findings of published literature [13,14,15] and agrees with studies that attribute a higher risk of TDIs to gender and age. Authors have postulated that males have a greater propensity towards contact sports and risky behavior than girls [6,16]. Recent studies, however, have argued a decline in gender disparities as females have developed similar athletic interests and are exposed to the same risk factors as boys in western society [17]. This is confirmed by Glendor et al., who argue that the frequency of TDIs increases due to types of activity and behavioral factors rather than gender [10]. It aligns with the fact that in both genders, TDIs were most frequently caused by falling (male: n = 189, 55.3%; female: n = 176, 74.6%). Many other studies have previously identified falling as the main cause of dental trauma [18,19]. With falling being the predominant cause of TDIs, the frequency of TDI occurrence on weekdays and weekends is expected to be similar. The presented data confirm this assumption, as the frequencies of TDI occurrence only show minimal variations in regard to the day of the week. TDI occurrence was higher on Fridays and Saturdays. As other recent studies have shown similar results, the relative likelihood of a TDI occurrence being higher from April to November may be linked to the fact that outdoor activities entailing higher risks of falling accidents increase in spring and summer [13,20].

Most patients initially sought initial treatment at the Department of Emergency Dental Care at the University Dental Clinic of Vienna (n = 382, 66.1%) during clinic hours. This confirms a rather high awareness level of patients of the presented patient collective to consult specialized dental departments rather than common emergency departments in general hospitals for TDIs. With regard to clinic arrival time following dental trauma, 66.0% (n = 356) sought treatment within the first 24 h after TDI. After one week, 89.1% of patients with TDIs (n = 480) had presented at the clinic. This emphasizes the importance of decentral dental care units within the appropriate distance. As TDIs fairly evenly occur on weekdays as well as on weekends, the opening hours of these support units should be extended to weekends. 

Evaluating the pattern of affected teeth, the results show that upper central incisors were the most frequently injured teeth (63.5%, n = 719). This is coherent with international data [2,14,15,21]. Due to their anterior position, upper incisors are more prone to be affected in traumatic incidents [17]. Additional risk factors include insufficient lip closure, an overjet over 3 mm and protrusion of upper anterior teeth [10,22,23]. In contrast, lower incisors are more likely to be protected from TDI by the lower lip and upper incisors. In this study only 6.7% of TDIs occurred in lower teeth. 

Based on the analysis of the presented data, fractures were the most common injury type (53%, n = 596), with uncomplicated crown fractures (without pulp exposure) being the most prevalent subtype (64.9%, n = 387). This confirms international trends [1,24]. However, the presented result is higher than the average observed by Faus-Matoses et al. (43.2%). Moreover, Rouhani et al. observed crown fractures in only 37% of cases, whereas luxation injuries dominated 46.1% in an Iranian school [18]. On the other hand, Bilder et al. observed very high values in uncomplicated crown fractures with respect to enamel fractures in 91.3% of cases [25]. The wide range of incidence rates reflects that the grounds for their comparison in reference to different injury types and their subgroups are often not satisfactory as varying classification systems are applied in different studies [26]. Compared to the results of Yeng. et al. (1.9%) [11] and Alhaddad et al. (1.85%) [13] avulsions were observed in much higher frequency in this study (13.6% of all dislocations). 

Evaluating the most common locations of the traumatic incident, the findings of the study reveal that in 79.4% (n = 459) of cases, TDI occurred at home. This is consistent with the results of a literature review by Bastone et al. that identified accidents within and around the home as the major source of TDIs [24]. In comparison to the observations by Rouhani et al. (46.8%) [18] and Alhaddad et al. (46.47%) [13], the results of this survey are much higher. Accidents during leisure activities, including sports, were observed in 9.5% (n = 55) of cases. Once more, this underlines the essential role of the previously mentioned behavioral characteristics in risk assessment. An example of measures reducing the risk and incidence of dental trauma during sports is the use of protective athletic appliances such as mouthguards. For instance, Ilia et al. reported a significant risk reduction for complications of TDIs in rugby union players in Australia after using mouthguards [27]. The results of Green’s study on the role of mouthguards in preventing and reducing sports-related traumas confirm the beneficial effect of this oral appliance [28]. The use of mouthguards can be especially recommended for people with oral risk factors such as class II occlusion and overjet over 3 mm. The fact that only 5.2% (n = 30) of TDIs were caused by traffic may be interpreted as a testament to the effectiveness of traffic preventive measures throughout the past decades in Austria.

This study’s findings should be framed by acknowledging its limitations. The analysis included a cohort of 578 patients that presented during opening hours on weekdays and weekends at the Department of Emergency Dental Care at the University Dental Clinic of Vienna. Dental trauma that presented with local dentists or other emergency units was not evaluated. A multicenter approach, including local dental offices and other emergency units, is strongly recommended in order to acquire more comprehensive data for TDIs in Vienna. It must also be noted that treatment outcomes were not evaluated in the present study. Furthermore, the comparison of incidence and prevalence in reference to different injury types with other studies, however, proved difficult as varying classification systems were applied. Combined injuries are often not examined as a separate entity. This highlights the importance of epidemiology and the study of patterns and causes of TDIs at a national and international population level on the basis of a standardized approach to reporting, classification, and methodology. Hence, a multicenter approach with the implantation of standardized classification protocols is recommended in order to gain a more comprehensive analysis of incidence, prevalence, and patterns of TDIs in permanent teeth in Austria, develop national references and explore regional differences. Standardized documentation in combination with the installation of prospective databases for initial assessment, treatment, and follow-up of treatment outcomes is strongly advised. 

## 5. Conclusions

This study presents the first research on dental trauma in permanent teeth in Vienna, Austria. By large, the results confirm previously published international trends. The high incidence of dental trauma leads to its frequent occurrence in daily clinical practice worldwide. As dentists are often challenged by their accurate diagnosis and treatment, further education and training in dental traumatology are vital in promoting beneficial treatment outcomes in the future. The establishment of a regional network of trained dentists would be favorable. In this regard, online introductions, such as the dental trauma guide, can be useful tools. Furthermore, the city’s approach to decentralized care structures should be continued, and the installation of more decentral support units and immediate dental care facilities within appropriate distances offering standardized documentation and treatment protocols should be encouraged.

## Figures and Tables

**Table 1:** Classification of dental trauma used in the present study [9].

Type of Trauma	Abbreviation
**Fracture** Enamel infraction Crown fracture With pulp exposure No pulp exposure Crown-root fracture Root fracture **Dislocation** Concussion Subluxation Lateral dislocation Intrusion Extrusion Avulsion **Combined injuries** Fractures and dislocations simultaneously occurring within the same tooth at the same time	**F** EF CFP CFX CRF RF **D** CCS LOS LDL INT EXT AVU **C**

**Table 2:** Frequency of TDI occurrence per weekday.

Day of the Week	n
Monday	56
Tuesday	58
Wednesday	70
Thursday	73
Friday	101
Saturday	114
Sunday	81

**Table 3:** Distribution of injury type.

Type of Trauma	Classification	n	%
Fracture		596	53
	EF	52	8.7
	CF	387	64.9
	CFP	92	15.4
	CRF	48	8.1
	RF	28	4.7
Dislocation		382	34
	CCS	83	21.7
	LOS	105	27.5
	LDL	102	26.7
	INT	15	3.9
	EXT	23	6.0
	AVU	52	13.6
Combined injury		154	14
	EF	37	24.0
	CF	98	63.6
	CFP	10	6.5
	CRF	2	1.3
	RF	12	7.8
	CCS	48	31.2
	LOS	50	32.5
	LDL	38	24.7
	INT	5	3.2
	EXT	4	2.6
	AVU	6	3.9

**Table 4:** Incidence matrix.

	EF	CF	CFP	CRF	RF	CCS	LOS	LDL	INT	EXT	AVU
**EF**	89	12	3	0	2	18	6	13	0	0	0
**CF**	12	485	0	0	3	33	36	17	4	2	4
**CFP**	3	0	102	0	1	1	5	1	1	1	0
**CRF**	0	0	0	50	0	0	2	0	0	0	0
**RF**	2	3	1	0	40	0	1	5	0	1	3
**CCS**	18	33	1	0	0	131	3	0	0	0	0
**LOS**	6	36	5	2	1	3	155	0	1	0	0
**LDL**	13	17	1	0	5	0	0	140	0	0	0
**INT**	0	4	1	0	0	0	1	0	23	0	0
**EXT**	0	2	1	0	1	0	0	0	0	30	0
**AVU**	0	4	0	0	3	0	0	0	0	0	58

**Table 5:** Accident and injury types of TDI.

Accident Type	Gender						Injury Type					
	Total		Male		Female		D		F		C	
	n	%	n	%	n	%	n	%	n	%	n	%
Punching	137	23.7	96	28.1	41	17.4	102	39.4	119	45.9	38	14.7
Falling	365	63.1	189	55.3	176	74.6	236	31.8	404	54.4	103	13.9
Collision accidents	17	2.9	14	4.1	3	1.3	11	35.5	19	61.3	1	3.2
Not known	59	10.2	43	12.6	16	6.8	33	33.3	54	54.5	12	12.1

## Data Availability

The data presented in this study are available on request from the corresponding author.
